# A narrative synthesis of research with 5-MeO-DMT

**DOI:** 10.1177/02698811211050543

**Published:** 2021-10-19

**Authors:** Anna O Ermakova, Fiona Dunbar, James Rucker, Matthew W Johnson

**Affiliations:** 1 Beckley Psytech, Beckley, UK; 2 Psychedelic Trials Group, Centre for Affective Disorders, Institute of Psychiatry, Psychology and Neuroscience, King’s College London, London, UK; 3 Department of Psychiatry and Behavioral Sciences, School of Medicine, Johns Hopkins University, Baltimore, MD, USA

**Keywords:** 5-methoxy-*N*, *N*-dimethyltryptamine, 5-MeO-DMT, classic psychedelic, hallucinogen, tryptamine

## Abstract

**Background:**

5-Methoxy-*N,N*-dimethyltryptamine (5-MeO-DMT) is a naturally occurring, short-acting psychedelic tryptamine, produced by a variety of plant and animal species. Plants containing 5-MeO-DMT have been used throughout history for ritual and spiritual purposes. The aim of this article is to review the available literature about 5-MeO-DMT and inform subsequent clinical development.

**Methods:**

We searched PubMed database for articles about 5-MeO-DMT. Search results were cross-checked against earlier reviews and reference lists were hand searched. Findings were synthesised using a narrative synthesis approach. This review covers the pharmacology, chemistry and metabolism of 5-MeO-DMT, as well epidemiological studies, and reported adverse and beneficial effects.

**Results:**

5-MeO-DMT is serotonergic agonist, with highest affinity for 5-HT_1A_ receptors. It was studied in a variety of animal models, but clinical studies with humans are lacking. Epidemiological studies indicate that, like other psychedelics, 5-MeO-DMT induces profound alterations in consciousness (including mystical experiences), with potential beneficial long-term effects on mental health and well-being.

**Conclusion:**

5-MeO-DMT is a potentially useful addition to the psychedelic pharmacopoeia because of its short duration of action, relative lack of visual effects and putatively higher rates of ego-dissolution and mystical experiences. We conclude that further clinical exploration is warranted, using similar precautions as with other classic psychedelics.

## Introduction

Plant- and fungi-based psychedelics have been used for centuries for healing or ritual purposes (Schultes and Hofmann, 1980), and there is an active culture of self-medication with psychedelics for mental health (Carhart-Harris and Nutt, 2010). The classical psychedelic drugs were investigated extensively in psychiatry before they were placed in Schedule I of the UN Convention on Psychotropic Substances 1971 (United Nations Convention on Psychotropic Substances, 1971) and resulted in significant barriers to research and drug development with them (Johnson et al., 2008; Rucker et al., 2018; Weston et al., 2020).

Over the past two decades, research has resumed and encouraging early phase clinical trials assessing psilocybin-assisted psychotherapy have been reported in unipolar mood disorders and anxiety (Carhart-Harris et al., 2016, 2018; Griffiths et al., 2016; Grob et al., 2011; Ross et al., 2016) and substance use disorders (Bogenschutz et al., 2015, 2018; Johnson et al., 2014; Noorani et al., 2018).

The duration of action of classical psychedelics varies considerably. After oral ingestion, the subjective effect of lysergic acid diethylamide (LSD) lasts approximately 12 h (Holze et al., 2019), while psilocybin lasts approximately 6 h (Hasler et al., 2004). Short-acting psychedelics may have therapeutic benefit (Nutt et al., 2020). Several survey studies have examined reports of addiction recovery prompted by the use of dimethyltryptamine (DMT) (Garcia-Romeu et al., 2019; Johnson et al., 2017). If efficacious, an advantage of short-acting psychedelics may be lower treatment costs. This may allow wider delivery of treatment, if clinical trial data supports licensing.

### History of discovery

5-Methoxy-*N,N*-dimethyltryptamine (5-MeO-DMT) is a short-acting serotonergic psychedelic that was first synthesised in 1936 (Hoshino and Shimodaira, 1936) and later isolated from *Dictyoloma incanescens* in 1959 (Pachter et al., 1959). Subsequently, 5-MeO-DMT has been found in a large number of plants (reviewed in Trout, 2007), notably *Anadenanthera, Phalaris* and *Virola* spp. (Rätsch, 2005; Schultes et al., 2001; Schultes and Hofmann, 1980). 5-MeO-DMT is found in fungi *Amanita citrina* and *Amanita porphyria* (Tyler and Gröger, 1964), as well as the gland secretions of the Sonoran Desert toad *Incilius* (formerly *Bufo*) *alvarius* (Erspamer et al., 1967; Uthaug et al., 2019; Weil and Davis, 1994) and in mammals (Barker et al., 2012; Beaton and Morris, 1984).

### Occurrence in nature

5-MeO-DMT is likely to be endogenously produced in humans, as it has been detected in blood, urine and cerebrospinal fluid (Christian et al., 1975; Corbett et al., 1978; Guchhait, 1976; Heller et al., 1970; Narasimhachari et al., 1971a, 1971b; Riceberg and Vunakis, 1978; Smythies et al., 1979; Tanimukai, 1967, Tanimukai et al., 1970), although several studies contradict this finding (Forsström et al., 2001; Himwich et al., 1972; Huszka et al., 1976; Narasimhachari et al., 1972, 1974). Pooling these studies together, 5-MeO-DMT was detected in urine of 2 out of 113 individuals, in blood of 20 out of 39 individuals and in cerebrospinal fluid of 40 out of 136 individuals. However, it is important to note that only the two later studies (Corbett et al., 1978; Smythies et al., 1979) used mass spectrometry, while the older studies used less reliable methods. The physiological role of 5-MeO-DMT is unknown and more research is needed to definitively answer if, when and where 5-MeO-DMT is endogenously produced.

### Traditional use

Indigenous peoples of South America have used 5-MeO-DMT containing plants for thousands of years (Pochettino et al., 1999; Torres and Repke, 2006). Snuffs from the beans of *Anadenanthera peregrina* (called *cohoba, yopo*) are prepared in northern South America, although the use of this plant in pre-Colombian times has been documented as far as the West Indies (Schultes and Hofmann, 1980). In central and southern parts of South America, snuffs called *vilca, huilca* and *cibil* produced from *A. colubrina* are used. Many species of *Virola* trees (e.g. *V. theiodora, V. calophylla, V. elongata*) are utilised by the indigenous peoples in the Amazon region (Schultes et al., 2001). 5-MeO-DMT is present in plants that are sometimes used as constituents in ayahuasca (Holmstedt et al., 1980).

The popularity of toad secretions is a fairly recent phenomenon traceable to the publication of a booklet by Albert Most (1984). There is no conclusive historical evidence for the indigenous use of *Incilius alvarius* toads for their psychoactive properties prior to this (Ott, 1996).

### Legal status

5-MeO-DMT was included in the Schedule 1 Controlled Substance Act in the United States of America in 2009 (Drug Enforcement Administration (DEA), Department of Justice, 2010) and is a controlled substance in the United Kingdom (Home Office, 2019), Australia (Federal Register of Legislation, 2016), New Zealand (New Zealand Legislation, 2021) and several other countries. It is not listed by the United Nations Convention on Psychotropic Substances, and in many countries, including Canada, this substance is not controlled (Canada Justice Laws, 1996).

Epidemiological surveys suggest increasing non-medical use of 5-MeO-DMT, with users often reporting improvements in outcomes relating to mental health (Davis et al., 2018; Uthaug et al., 2019, 2020b).

This narrative review of published 5-MeO-DMT research aimed to synthesise the available literature and provide a comprehensive overview of the pre-clinical and safety data. It is considered timely and important because interventional clinical trials with this compound are being initiated (Clinicaltrials.gov, 2019, 2021a, 2021b).

## Methods

References for this article were identified via a search of PubMed from January 1965 to October 2020 using the terms ‘5-methoxy-N,N-dimethyltryptamine’ or ‘5-MeO-DMT’ and other variations on the chemical name (for full search terms, see Supplementary Material). Papers in English, Russian or Spanish were included, representing the fluent language proficiencies of the authors. The PubMed search was supplemented by additional articles, which were identified during the review of the bibliographies from the papers sourced through PubMed. References were then selected on the basis of relevance to the content of review.

**Figure 1.**
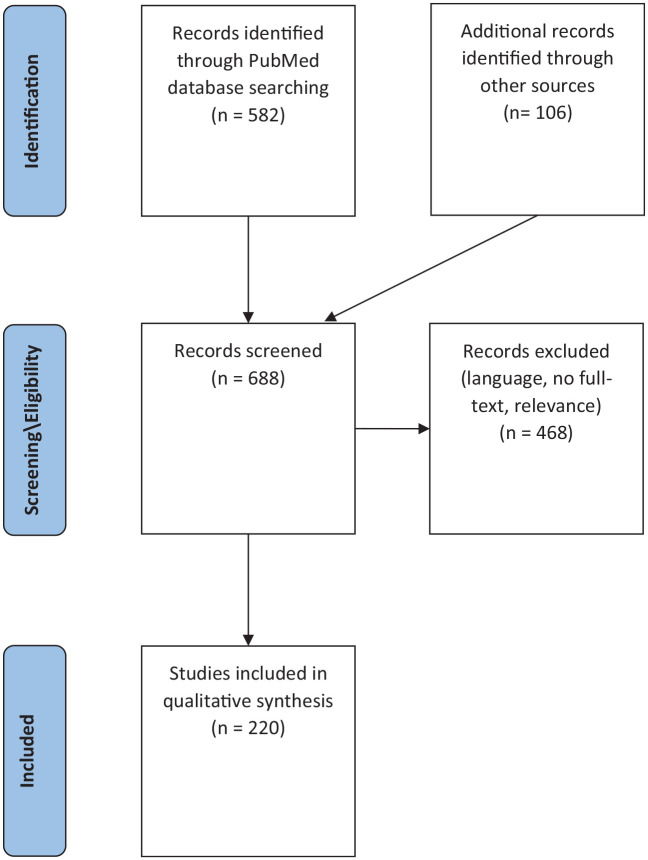
PRISMA flow diagram.

As this review aims to inform future clinical research, we excluded studies on the chemical synthesis or forensic detection of 5-MeO-DMT, articles identifying 5-MeO-DMT in plants and others that were not providing novel information about 5-MeO-DMT aside from its use at 5-HT agonist. The number of sources we identified, screened and included/excluded can be found in Figure 1.

## Description of studies/topics

### Chemical properties

2-(5-methoxy-1H-indol-3-yl)-*N,N*-dimethylethanamine (5-methoxy-*N,N*-dimethyltryptamine, abbreviated to 5-MeO-DMT) is a tryptamine alkaloid, an aromatic ether and a tertiary amine with a molecular weight of 218.298 g/mol and the chemical formula C_13_H_18_N_2_O. As the freebase, 5-MeO-DMT is a white solid with a melting point of 69.5°C. Water solubility is >32.7 μg/mL (PubChem, 2021).

### In vitro pharmacology

5-MeO-DMT is a non-selective serotonin (5-HT) receptor agonist, with affinity to other receptors, as well as to serotonin and norepinephrine transporters (Halberstadt et al., 2012; PDSP Database, 2021; Ray, 2010; see Table 1). 5-MeO-DMT is a weak 5-HT reuptake inhibitor but has no appreciable effects on monoamine release nor on noradrenaline or dopamine reuptake (Berge et al., 1983; Blough et al., 2014; Nagai et al., 2007). 5-MeO-DMT has high affinity for a range of 5-HT receptors, particularly (inhibition constant [K_i_] < 100 nM) at cloned human 5-HT_1A_, 5-HT_1B_, 5-HT_1D_, 5-HT_6_ and 5-HT_7_ receptor subtypes. High affinity is seen for the 7-transmembrane G-protein-coupled 5-HT receptors, with no affinity for the ion-channel 5-HT_3_ receptor. 5-MeO-DMT’s binding affinity to sigma receptors is >10,000 nM, although one study indicated that 5-MeO-DMT can impact immune responses in human monocyte-derived dendritic cells via σ-1 (Szabo et al., 2014).

**Table 1.** Receptor binding profiles for 5-MeO-DMT.

Binding sites	Binding affinity, Ki (nM) Ray (2010)	Binding affinity, Ki (nM) Halberstadt et al. (2012)	Binding affinity, Ki (nM)PDSP Ki database
Serotonin (5-HT) receptors			
5-HT_1A_	1.9	3.0	
5-HT_1B_	74	14	351
5-HT_1D_	6.3	2.3	
5-HT_1E_	360.2	376	
5-HT_2A_	2011	907	14
			390
			207
			600
			616
			617
			620
5-HT_2B_	3884	36	1300
5-HT_2C_	538	418	87.1
			100
5-HT_5A_	276.6	505	
5-HT_6_	35.2	6.5	
5-HT_7_	3.9	4.5	
Dopamine receptors			
D_1_	79.5	>10,000	
D_2_	3562	>10,000	
D_3_	497.6	>10,000	
D_4_	3120	>10,000	
D_5_	>10,000	>10,000	
Norepinephrine receptors			
α_1A_	>10,000	4373	
α_1B_	>10,000	2188	
α_2A_	1890	938	
α_2B_	2640	430	
α_2C_	508.1	206	
β_2_	>10,000	2679	
Other receptors and transporters			
σ-1	>10,000	>10,000	
σ-2	>10,000	3689	
H_1_	ND	7580	
Serotonin transporter protein (SERT)	2032	3603	
Dopamine active transporter (DAT)	>10,000	>10,000	
Norepinephrine transporter (NET)	2859	>10,000	

The raw Ki data is from Supplementary Table S2, Ray (2010) or Table 1, Halberstadt et al. (2012), both based on cloned human receptors in cell lines. 5-MeO-DMT also binds to the trace amine-associated receptor 1 (TAAR1), but the Ki is not provided (Wallach, 2009). 5-MeO-DMT bound to the following sites with Ki values >10,000 nM: 5-HT3, Ca^2+^ channels, β1, β3, DOR, MOR, KOR, EP3, EP4, GABAA, H2, H3, H4, M1, M2, M3, M4, M5 (Halberstadt, 2012). Additionally, we present older data, based on rat or pig brain homogenates, retrieved from PDSP Ki database (Roth et al., 2000) in January 2021 (https://pdsp.unc.edu/databases/kidb.php).

Receptor binding profiles based on human cloned receptors in cell lines presented by Halberstadt et al. (2012) and Ray (2010) are shown in Table 1. Although their findings are similar for the 5-HT receptors, they differ for others; further research is required to resolve these discrepancies.

Radioligand binding studies show that 5-MeO-DMT has about 300-fold selectivity for the 5-HT_1A_ (3 ± 0.2 nM) versus 5-HT_2A_ (907 ± 170 nM) receptor subtypes (Halberstadt et al., 2012). Other receptor types have not been studied as extensively (Halberstadt and Geyer, 2011).

More research is needed to resolve the full receptor binding profile of 5-MeO-DMT and understand the functionally selective pharmacology at 5-HT_2A_ and other receptors. Precise changes in receptor conformation result in different signalling cascades with different effects (e.g. behavioural or gene expression) (Urban et al., 2007). Put another way, every ligand has its own signalling signature, which may (or may not) be similar to the endogenous ligand.

For example, hallucinogenic and non-hallucinogenic 5-HT_2A_ agonists differentially activate second messenger pathways (González-Maeso et al., 2007). Kurrasch-Orbaugh et al. demonstrated that 5-MeO-DMT activated phospholipase A_2_ (PLA2) signalling 13-fold more than phospholipase C (PLC) signalling (Kurrasch-Orbaugh et al., 2003). Added to this, β-arrestins are scaffolding proteins that can attenuate or facilitate G-protein-coupled receptor activity by, for example, receptor internalisation or the formation of heteroreceptors (Gurevich and Gurevich, 2004). Schmid and Bohn demonstrated that the actions of 5-HT require the β-arrestin-2 signalling pathway and activation of protein kinase B, while 5-MeO-DMT activates signalling cascades independent of β-arrestin-2 (Schmid and Bohn, 2010). Blough et al. (2014) confirmed this observation, demonstrating a 100-fold difference in potency for the G-protein-coupled compared to the β-arrestin signalling pathway for 5-MeO-DMT. Overall, the functional selectivity of exogenous versus endogenous ligands for receptors is highly complex, but likely important in understanding their observable effects.

### Pharmacokinetics

The pharmacokinetics of 5-MeO-DMT has been studied in mice (Jiang et al., 2013, 2015, 2016a; Shen et al., 2010a, 2011b, 2011) and rats (Halberstadt, 2016; Sitaram et al., 1987a, 1987b, 1987c, 1987d).

#### Absorption

The maximum concentration (*C*_max_) in plasma is reached after 5–6 min following an intraperitoneal (IP) injection, and the terminal half-life (*t*_1/2_) is 12–19 min in mice (Shen et al., 2009). A similar profile is seen in rats, with *C*_max_ = 5–10 min and *t*_1/2_ = 6–16 min (Sitaram et al., 1987a, 1987d).

#### Tissue distribution and protein binding

5-MeO-DMT is lipid-soluble (3.30 oil/water partition coefficient) and readily crosses the blood–brain barrier (BBB) (Gessner et al., 1968). 5-MeO-DMT distributes to the liver, kidneys and brain similarly in different animal models: rabbit, rat and mouse (Berger et al., 1978; Sitaram et al., 1987c, 1987d; Sitaram and McLeod, 1990). Brain concentrations of 5-MeO-DMT in the rat are 1.7-fold higher compared to plasma 45 min after IP injection, with highest concentrations in the cortex, thalamus, hippocampus, basal ganglia, medulla, pons and cerebellum (Barker et al., 2001; Sitaram et al., 1987c). In the mouse brain, 5-MeO-DMT distributes to the cortex, hippocampus, hypothalamus and striatum after IP administration (Shen et al., 2010b).

#### Metabolism and excretion

Shen et al. (2011) demonstrated that the pharmacokinetics of 5-MeO-DMT is non-linear for both IP and intravenous (IV) administration of high doses in mice. The estimated parameters for both IP and IV routes are as follows: maximum rate of reaction (V_max_), Michaelis constant (K_m_), clearance (CL) and additional clearance (CL_CYP2D6_) values are 2.76 mmol/min per kg, 13.2 mM, 0.21 min^−1^ kg^−1^ and 0.0256 L/min per kg, respectively. The CL_CYP2D6_ value represents the additional linear clearance of 5-MeO-DMT from the central compartment that is dependent on CYP2D6 protein.

**Figure 2.**
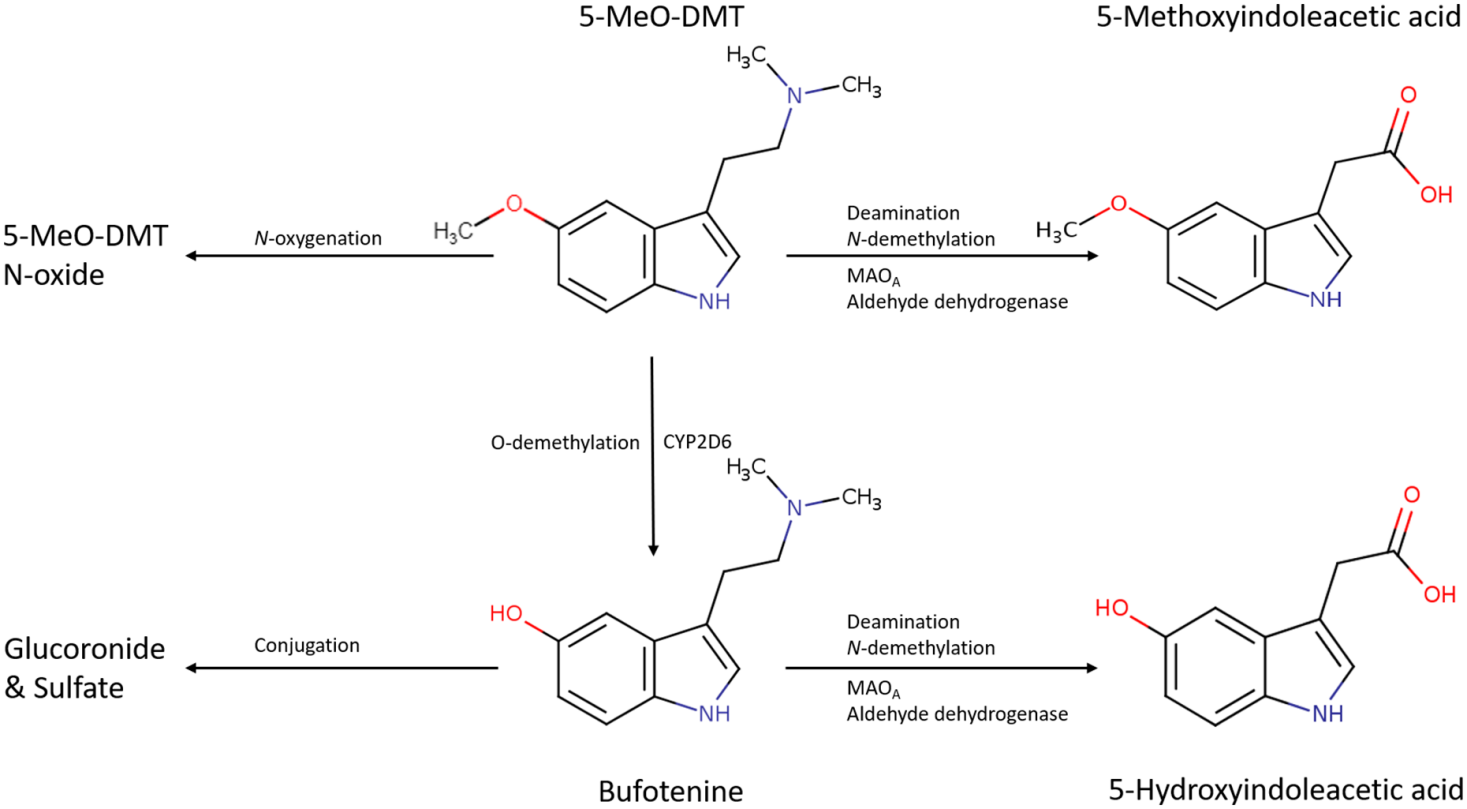
Metabolism of 5-MeO-DMT.

5-MeO-DMT is extensively metabolised through oxidative deamination catalysed by monoamine oxidase A (MAO_A_). O-demethylation, N-demethylation and N-oxygenation are involved to a much smaller extent (Sitaram and McLeod, 1990). Metabolic studies performed in rats showed that 5-methoxyindoleacetic acid (5-MIAA) is the main urinary metabolite of 5-MeO-DMT (54%), followed by 5-hydroxy-*N,N* dimethyltryptamine glucuronide (23%), 5-hydroxyindoleacetic acid (5-HIAA, 14%) and bufotenine (9%) (Agurell et al., 1969; Ahlborg et al., 1968; Sitaram et al., 1987d; Squires, 1975; Suzuki et al., 1981; Yu et al., 2003; see Figure 2).

When doses of 10 or 20 mg/kg of 5-MeO-DMT (IP and IV) are administered to mice, a 50% decrease in systemic clearance is observed, indicating that MAO_A_-mediated metabolism becomes saturated. This non-linearity is also reflected in corresponding increases in brain concentration of 5-MeO-DMT (Shen et al., 2010b, 2011).

The extent of O-demethylation depends on the genetic variant of the cytochrome P450 2D6 enzyme (CYP2D6). This enzyme mediates production of the psychoactive metabolite bufotenine from 5-MeO-DMT (Shen et al., 2010b).

### In vivo pharmacology

Studies of 5-MeO-DMT have been conducted in mice, rats, gerbils, hamsters, guinea pigs, rabbits, goldfish, cats, dogs, sheep, pigs and primates. The most common route of administration for rodents was subcutaneous or intraperitoneal. For a summary of the doses, routes of administration and behavioural effects by species, see Tables 2 and 3.

**Table 2.** Doses and routes of administration in different species.

Species	References	Threshold dose	Effective dose	High dose	LD_50_
Goldfish	Abramson et al. (1979)	NA	5 µg IC for a 1.5–3 g fish	NA	NA
Mouse*	Jiang et al. (2016a, 2016b); Winter et al. (2011); Martin et al. (1985); Moser and Redfern (1985); Gillin et al. (1976); Ho et al. (1970); Benington et al. (1965)	NA	0.3–5 mg/kg IP, SC	>8 mg/kg IP, IV >32 mg/kg IP toxicity	75–115 mg/kg IP 48 mg/kg IV 113 mg/kg SC 278 mg/kg O
Rat*	Halberstadt et al. (2008, 2012); Krebs-Thomson (2006); Critchley and Handley (1987); Winter and Petti (1987); Gudelsky et al. (1986); Tricklebank et al. (1985); Trulson and MacKenzie (1981); Bedard and Pycock (1977)	<0.1 mg/kg IP, SC	0.5–2 mg/kg IP, SC	>3 mg/kg IP	NA
Gerbil	Eison and Wright (1992)	0.5 mg/kg SC	NA	8 mg/kg SC	NA
Guinea-pig	Evenden (1994); Nielsen (1998)	NA	1–10 mg/kg SC	NA	NA
Hamster	Richter and Löscher (1995)	NA	1–4 mg/kg IP	>5 mg/kg IP	NA
Rabbit	Romano et al. (2010)	NA	0.29 mg/kg	NA	NA
Cat	Trulson and Jacobs (1979); Benington et al. (1965)	0.025 mg/kg IM	0.25–0.5 mg/kg IM 0.1–0.3 IV	>1–5 mg/kg IM, IV,	15 mg/kg IM
Dog	Gessner et al. (1961)	NA	0.1 mg/kg IV	NA	NA
Sheep	Bourke et al. (1990); Bourke et al. (1988); Gillin et al. (1976); Gallagher et al. (1964)	0.02 mg/kg IV >18 mg/kg O	0.1–0.7 mg/kg IV 40 mg/kg O	>1 mg/kg IV >40 mg/kg O	1–5 mg/kg IV 1–2 mg/kg SC 85 mg/kg O
Pig	Löscher et al. (1990)	NA	0.5–1.8 mg/kg IV	NA	NA
Grivet monkey	Nielsen (1985)	NA	0.45 mg/kg SC	NA	NA
Stumptail macaque monkey	Schlemmer and Davis (1981, 1981); Schlemmer et al. (1977)	0.05 mg/kg IM	0.1–0.25 mg/kg IM	NA	NA
Rhesus monkey	Gillin et al. (1976)	>0.1 mg/kg IV	0.25 mg/kg IV	8–16 mg/kg IV	NA
Human	Erowid (2021); Metzner (2013); Shulgin and Shulgin (1997); Ott (2001); Davis et al. (2018); Uthaug et al. (2020a)	1–2 mg S 3–5 mg IN 0.25 mg IV	2–10 mg S 5–15 mg IN 10 mg SL 10–30 mg O 0.5–2 mg IV 1.4–10 mg IM	10–20 mg S 10–25 mg IN >30 mg O >2 mg IV	NA

IC: intracranial; IM: intramuscular; IN: intranasal; IP: intraperitoneal; IV: intravenous; MAOI: monoamine oxidase inhibitor; NA: information not available; O: oral; S: smoked or vapourised; SC: subcutaneous; SL: sublingual.

Minimal (or threshold) dose is defined as the dose after which any difference in behaviour or physiology compared to baseline is observed. Effective dose is defined in a similar way to ED_50_ and reliably produces hallucinogenic-like and other characteristic behavioural effects in animal models. High dose is the one leading to marked serotonin syndrome or other serious adverse effects. Note that these dose ranges are an approximation, depend on particular behaviour/task, and in some species based on only one or two studies and drug administrations. The species differences could be due to the pharmacokinetics and metabolism differences, as well as the physiology of the specific animal models. It is also likely that direct mg/kg comparison is not appropriate across species and interspecies scaling factor is necessary for the meaningful comparison.

* Representative references, selecting studies containing multiple doses.

**Table 3.** Behavioural effects of 5-MeO-DMT in different species.

Species	Effects	References
Cotton boll weevil (*Anthonomus grandis*)	Potent anti-feeding effect	Miles et al. (1987)
Goldfish (*Carassius auratus*)	Surfacing behaviour, which is a characteristic hallucinogen response in fish	Abramson et al. (1979)
Mouse* (*Mus musculus*)	• Mice discriminate 5-MeO-DMT from saline • Dose-dependent increase in the head-twitch response. Head-twitch response shows circadian variation • Reduction in locomotor activity and in investigatory behaviour • Increased latency to feed in the novel environment • Inhibition of isolation-induced aggression • Inhibition of memory retention in conditional avoidance task • Facilitation of memory retention in active avoidance task by lower, but not higher doses • Straub tail, forepaw threading, twitching, flat-body posture, hindlimb abduction, tremors • Reduced sensitivity to pain (increased tail flick response latencies)	Jiang et al. (2016a, 2016b); Winter et al. (2011); Halberstadt et al. (2011); Van den Buuse et al. (2011); Duvvuri et al. (2009); Pavone et al. (1993); Sánchez et al. (1993); Eide and Tjølsen (1988); Quartermain et al. (1988); Martin et al. (1985); Moser and Redfern (1985); Singleton and Marsden (1981); Benington et al. (1965)
Rat* (*Rattus norvegicus domestica*)	• Rats discriminate 5-MeO-DMT from saline, but not from other classic psychedelics • Dose-dependent inhibition of locomotor activity, reduction in investigatory behaviour (increased fear of the open spaces and novel objects) • Dose-dependent increase in forepaw treading, flat-body posture, Straub tail response, hindlimb abduction, tremor, head-twitch response–no sex differences in these effects in response to 5-MeO-DMT • Medium doses: ‘wet-dog’ shakes, head shakes, lower lip retraction • Increased latency to feed in the novel environment (hyponeophagia) and reduced palatability of sucrose drink • High doses: severe tremors, shivering, biting of paws, convulsions, muscle spasms, rocking from side to side, compulsively biting grid floor/cage, walking backwards and abnormal gait, inhibit shock-elicited fighting, respiratory arrest • Non-linear effect on nociception, measured with tail flick latencies (enhanced at low doses, reduced at high doses) • Inhibition of conditioned avoidance, failure to orient towards ‘warning’ stimuli, deficit in aversive conditioning learning • Normal doses stimulate sexual behaviour (mounts, ejaculatory response, a decrease in the number of intromissions to ejaculation and in the ejaculation latency) in male rats • In neonatal rats, 5-MeO-DMT at normal doses had no effect in either 5- or 20-day-old pups. High dose produced increase in locomotion in 5-day-old pups, and hyperlocomotion, tremor, flattened body posture, forepaw threading and head weaving in 20-day-old pups • Chronic administration: tolerance develops to some drug effects, but only with very frequent drug administration (twice/per day or every 30 min)	Halberstadt et al. (2008, 2012); Krebs-Thomson et al. (2006); Winter et al. (2000); Ahlenius and Larsson (1991); O’Hare et al. (1991); Berendsen et al. (1989); Critchley and Handley (1987); Winter and Petti (1987); Dickinson and Curzon (1986); Rényi (1986a, 1986b); Sills et al. (1985); Tricklebank et al. (1985); Trulson et al. (1985); Archer et al. (1982); Shephard and Broadhurst (1982); Berge (1982); Berge et al. (1980); Walters et al. (1978); Bedard and Pycock (1977); Gillin et al. (1976); Grahame-Smith (1971); Ahlborg et al. (1968); Gessner and Page (1962); Gessner et al. (1961)
Mongolian gerbil (*Meriones unguiculatus*)	• Reciprocal forepaw treading, reciprocal hindleg body scratch, hindleg abduction, body tremors and Straub tail	Eison and Wright (1992)
Guinea-pig (*Cavia porcellus*)	• Dose-dependent increase in the locomotor activity of naïve, unhabituated guinea pigs. Interestingly, there results are opposite to what is commonly observed in rats, that is, decrease in locomotor activity • At higher doses head jerking and whole-body myoclonic jerking. Flat-body posture, tremor and head twist or head shake	Evenden (1994) Nielsen (1998)
Syrian hamster (*Mesocricetus auratus*)	• At lower doses, the predominant effect was flat-body posture • Higher doses additionally induced hyperlocomotion and hindlimb abduction, plus salivation, ataxia and piloerection. Unlike in studies with other rodents, no forepaw threading was observed	Richter and Löscher (1995)
Rabbit (*Oryctolagus cuniculus domesticus*)	• 5-MeO-DMT produced head bobs but not body shakes	Romano et al. (2010)
Cat (*Felis catus*)	• Limb flicking, abortive grooming (starting the motion as if to groom, and stopping it), head shaking, staring, investigatory and hallucinatory-like behaviours similar to those produced by other psychedelics, except for the faster onset and shorter duration in case of 5-MeO • ‘Sham rage’ response, hissing, growling, withdrawal, salivation • Inhibition of conditioned avoidance response (i.e. no reaction to auditory stimulus associated with electric shock) • Chronic effects: no tolerance during once-daily administration	Trulson and Jacobs (1979); Benington et al. (1965)
Sheep (*Ovis aries*)	• Low doses: urination, tail, ear and lip twitching, lip licking, head shaking, agitation, pupil dilation, mild hind limb paresis and mild ataxia • Medium doses: chewing movements, salivation, head and body tremors, neck extension, hind limb paresis, ataxia, hypermetria, walking backwards or in circles, walking on the knees and intermittent periods of either sitting on the haunches of in sternal recumbency, moderate pelvic and thoracic limb paresis, disturbed equilibrium and laboured breathing • High doses: all of the previous clinical signs plus protracted periods of recumbency accompanied by vigorous attempts to get up, knuckling over in the fore fetlocks, mild cyanosis of the mucous membranes and mild respiratory distress, muscle rigidity, intermittent periods of reduced consciousness when the animals lay down, with necks extended, heads swaying, and eyes staring, tetanic spasms, acute respiratory and heart failure, death	Bourke et al. (1988, 1990); Gillin et al. (1976); Gallagher et al. (1964)
Pig (*Sus scrofa domesticus*)	• Grimacing, backward locomotion, blank stare, screams, head shakes, generalised tremor followed by lateral recumbency with muscle rigidity	Löscher et al. (1990)
Grivet monkey (*Chlorocebus aethiops*)	• 5-MeO-DMT substitutes completely for LSD in drug discrimination studies	Nielsen et al. (1985)
Stumptail macaque monkey (*Macaca arctoides*)	• Acute effects: increased submissive gestures and hyperactivity, a reduction in social grooming and other social behaviour, an increase in distancing from other monkeys, and increase in checking, limb jerks, body shakes. Animals appear alert and restless • Chronic effects: no tolerance after daily administration. With more frequent drug administration every 30 min for 9 h, and then 26 h later tolerance developed to limb jerks, body shakes and checking behaviour	Schlemmer and Davis (1981, 1986); Schlemmer (1977); Heinze et al. (1983)
Rhesus macaque monkey (*Macaca mulatta*)	• Medium doses: ataxia, decreased spontaneous movement and climbing, and unresponsiveness to salient external stimuli, slow nystagmoid movements and mydriasis, stringy salivation, jaw clenching, loss of motor coordination and diminished muscle tone • High doses: animals were comatose and could not be aroused • Chronic: no tolerance to 5-MeO-DMT with daily administration	Gillin et al. (1976)

LSD: lysergic acid diethylamide.

* For rats and mice, representative references are presented.

### Behavioural effects

The behavioural effects of 5-MeO-DMT have been best characterised in rodents and are similar to those of other classic hallucinogens, although rodent strain differences have been observed (Cazala and Garrigues, 1983; Gudelsky et al., 1985; Shephard and Broadhurst, 1983; Stoff et al., 1978). Rats quickly learn to discriminate 5-MeO-DMT from saline (Glennon et al., 1979, 1982a, 1982b; Spencer et al., 1987), but not from another classic psychedelic, including partial generalisation with a more selective 5-HT_2A, 2B_ and _2C_ agonist 2,5-Dimethoxy-4-methylamphetamine (DOM) (Glennon et al., 1979, 1980, 1982a, 1982b; Spencer et al., 1987; Winter et al., 2000; Young et al., 1982). The 5-MeO-DMT discriminative stimulus involves both 5-HT_1A_- and 5-HT_2A_-mediated components, although the latter plays a less important role as the discriminative stimuli induces by 5-MeO-DMT are diminished by 5-HT_1A_ antagonists (Schreiber and de Vry, 1993; Spencer et al., 1987; Winter et al., 2000). A signature behavioural response to 5-HT_2A_ receptor stimulation and a behavioural mode of hallucinogenic effect in rodents, the head-twitch, is induced by 5-MeO-DMT over a comparable dose range to 5-HT_1A_-mediated behaviours, is attenuated by selective 5-HT_2A_ receptor antagonists and is absent in 5-HT_2A_-knockout mice (Halberstadt, 2016; Halberstadt et al., 2011; Matsumoto et al., 1997).

Other behavioural effects of 5-MeO-DMT are predominantly 5-HT_1A_ mediated, although 5-HT_2A_ receptor activation is also involved (Berendsen et al., 1989; Eison and Wright, 1992; Halberstadt and Geyer, 2011; Krebs-Thomson et al., 2006; Lucki et al., 1984; Smith and Peroutka, 1986; Tricklebank et al., 1985). Activity at the 5-HT_2C_ receptor serves to modify some of the behavioural effects of hallucinogens (Halberstadt et al., 2011). 5-MeO-DMT dose dependently reduces locomotor activity, reduces investigatory behaviour but induces forepaw treading, flat-body posture, Straub tail response and hindlimb abduction. This appears to be mainly mediated through 5-HT_1A_ receptors, with some contribution of 5-HT_2A_ receptors (Bedard and Pycock, 1977; Castellanos et al., 2020; Eide and Tjølsen, 1988; Halberstadt, 2016; Halberstadt et al., 2008, 2011, 2012; Jiang et al., 2016b; Krebs-Thomson et al., 2006; Matsumoto et al., 1997; Matthews and Smith, 1980; Rigdon and Weatherspoon, 1992; Smith and Peroutka, 1986; Tricklebank et al., 1985; Van den Buuse et al., 2011). 5-MeO-DMT at high doses inhibits shock-elicited fighting in rats (Walters et al., 1978). 5-MeO-DMT at medium doses stimulates male sexual behaviour in rats (Ahlenius and Larsson, 1991; Kolbeck and Steers, 1992; Rényi, 1986a, 1986b). See Table 3 for the full list of behavioural effects in different animal models.

### Neurobiological effects

In a healthy volunteer field study evaluating EEG and psychedelic experience correlates, Acosta-Urquidi observed that smoked 5-MeO-DMT suppressed alpha frequencies acutely, followed by a rebound increase in alpha-power ~20 min post inhalation. The time course and intensity of the subjective experience correlated with the magnitude of the observed EEG effects (Acosta-Urquidi, 2015). Other effects were an emergent increase in the delta/theta power. The findings are broadly consistent with those from a DMT study (Timmermann et al., 2019).

Riga et al. (2016, 2014, 2018) have investigated the neuropharmacology of 5-MeO-DMT in various rodent models and propose that effects on medial prefrontal cortex (mPFC) oscillatory activity and cortico-thalamic coherence underpin its antidepressant-like effect. 5-MeO-DMT disrupted low-frequency mPFC oscillations in a similar way to other 5-HT_2A_-mediated classic psychedelics and decreased blood oxygen level–dependent (BOLD) responses in visual cortex (V1) and mPFC. The effects observed in both normal and 5-HT_2A_ knockout mice were reversed by a 5-HT_1A_ receptor antagonist, indicating the importance of 5-HT_1A_ receptors in the effects of 5-MeO-DMT (Riga et al., 2016, 2018). In rats, 5-MeO-DMT altered the frequency and pattern of firing of level V pyramidal neurons in mPFC and reduced the amplitude of low-frequency oscillations (Riga et al., 2014).

Winne et al. (2020) found that pre-treatment with 5-MeO-DMT prevented anxiety-like behaviour (measured in the open field test and elevated plus maze) and abnormal neural activity (increase in theta 2 and slow gamma oscillations in the hippocampus and mPFC) triggered by tinnitus in mice.

Lima da Cruz et al. demonstrated that 5-MeO-DMT increases neuronal progenitor cell proliferation and survival in the mouse hippocampus. A single dose of 5-MeO-DMT increased the number of progenitor cells in the dentate gyrus, which survived better and matured faster (i.e. had more complex dendrites and greater capacity for high-frequency firing) compared to those of saline-treated animals (Lima da Cruz et al., 2018).

Earlier studies examined the effects of 5-MeO-DMT on cat and rat neuron firing in the central and peripheral nervous systems. Generally, 5-MeO-DMT in cats produces a rapid, dose-dependent inhibition of 5-HT neuronal activity (Adrien and Lanfumey, 1986; Fornal et al., 1985, 1994; Heym et al., 1982; Jacobs et al., 1983; Kodama et al., 1989; Rasmussen et al., 1984; Trulson et al., 1984a, 1984b) and antiepileptic effects (Wada et al., 1992). 5-MeO-DMT increases the excitability of several types of spinal neurons, including motoneurons, and consequently influences the locomotor pattern as well as the reflex responsiveness in cats with severed spinal cords (Barbeau and Rossignol, 1990). In rats, 5-MeO-DMT dose dependently increases the activity of motoneurons through 5-HT_2_ receptors, but it has an inhibitory action on the pathway of the monosynaptic reflex (Yamazaki, 1992).

### Cardiovascular effects

Psychedelics may increase heart rate and blood pressure via the sympathomimetic effects of 5-HT_2A_ receptor agonism. 5-HT_1A_ agonists however, decrease blood pressure and heart rate via peripheral vasodilation and vagus nerve stimulation (Dabiré, 1991; Kaumann and Levy, 2006). In healthy anaesthetised dogs and cats, 0.1 mg IV 5-MeO-DMT had a triphasic effect on blood pressure; an immediate rapid fall, followed by a brisk rise and finally a more prolonged fall (Gessner et al., 1961). A modest biphasic blood pressure response, with initial dose-dependent increase followed by a decrease, accompanied by a slight decrease in heart rate has also been demonstrated in rats (Dabiré et al., 1987). Bradycardia was also observed in rhesus monkeys, but otherwise electrocardiography measures were normal with doses up to 8 mg/kg. However, it is important to note that in addition to the direct cardiovascular effects described above, there are also likely to be indirect, psychosomatic effects of anticipating or having an intense psychedelic experience.

5-MeO-DMT, like psilocybin, binds to 5-HT_2B_ receptors (Halberstadt et al., 2012). Some 5-HT_2B_ agonists are associated with valvular heart disease (Roth, 2007; Rothman et al., 2000). However, to date, no research studies link classic psychedelic use and valvular heart disease. Any potential toxicity would likely be dose and frequency dependent.

### Thermoregulatory effects

Stimulation of different 5-HT receptors can have opposing effects on thermoregulation: Hypothermia can be triggered by 5-HT_1A_ receptor agonists while 5-HT_2A_ stimulation can cause hyperthermia (Gudelsky et al., 1986a, 1986b). 5-HT_2A_ receptor-related vasoconstriction is thought to be a main effector site of serotonergic thermoregulation (Ootsuka et al., 2004). Using an experimental drug administration and mathematical pharmacokinetic/pharmacodynamic (PK/PD) model, Jiang et al. (2016b) demonstrated that 5-MeO-DMT induces transient hyperthermia in mice. However, another study showed that 3 mg/kg 5-MeO-DMT reduced tail-skin temperature in mice by 1.8°C (Eide and Tjølsen, 1988). In rats, 5-MeO-DMT has a non-linear effect on body temperature: at low (0.5–1.0 mg/kg) doses causing hypothermia but hyperthermia at high doses (3–10 mg/kg). The hyperthermic effect may be completely attenuated or even converted into hypothermia by the 5-HT_2A_ antagonist, ketanserin (Gudelsky et al., 1986a, 1986b). 5-MeO-DMT at 0.5–1.8 mg/kg also caused hyperthermia in pigs. Administration of higher doses to pigs genetically susceptible to malignant hyperthermia was fatal (Löscher et al., 1990).

### Effects on nociception

The analgesic effects of 5-MeO-DMT are also non-linear: Nociception in rats is enhanced after very low doses (1.6–25 µg) and then becomes biphasic at medium doses (hyperalgesia followed by analgesia at 50–100 µg) and reduced after higher doses (400 µg) of 5-MeO-DMT (Berge et al., 1980).

### Endocrine effects

5-MeO-DMT causes increased prolactin levels, dose dependently in both male and female rats (Carlsson and Eriksson, 1986; Meltzer et al., 1978; Seeman and Brown, 1985), although there is one report of a biphasic response, with initial increase followed by decrease (Simonovic and Meltzer, 1983). Repeated administration of 5-MeO-DMT (5 mg/kg, every 3 h for a total of four injections) potentiated its prolactin-releasing effect (Simonovic and Meltzer, 1979).

A prospective examination of 5-MeO-DMT inhalation in humans demonstrated that a single inhalation of 5-MeO-DMT increases cortisol levels in saliva (Uthaug et al., 2020b).

### Immunological effects

5-MeO-DMT can modulate immune responses in human primary immune cell cultures (Szabo et al., 2014). Treatment of immune-challenged, human monocyte-derived dendritic cells with 5-MeO-DMT resulted in a marked decrease in gene expression and secretion of various inflammatory cytokines and chemokines (IL-1β, IL-6, IL-8 and TNF-α), while strongly increasing the levels of the cytokine interleukin-10 (IL-10), an anti-inflammatory cytokine, mediated via the σ-1 receptor. In two different models, 5-MeO-DMT had strong immune modulating effects, with no impact on antibody production, immune homeostasis interleukins IL-4, IL-5 or T helper 2 cells. In a human study, a single inhalation of 5-MeO-DMT decreased the levels of circulating IL-6 (Uthaug et al., 2020b).

### Effects on gene expression

Dakic et al. (2017) studied the effects of 5-MeO-DMT on proteins in human brain organoids. Using mass spectrometry and shotgun proteomics, they identified more than 900 proteins (out of ~6700 sampled) differentially expressed after treatment with 5-MeO-DMT. These proteins impact anti-inflammatory effects, long-term potentiation, the formation of dendritic spines, microtubule dynamics and cytoskeletal reorganisation.

### Drug interactions

Jiang et al. (2013) examined 5-MeO-DMT interactions with MAO_A_ inhibitors. Coadministration of even a relatively low dose of harmaline (an inhibitor of monoamine oxidase) readily blocks MAO_A_-dependent elimination in mice, shifting 5-MeO-DMT metabolism to alternative pathways such as O-demethylation. This leads to a greater rate of conversion to bufotenine and significantly extends systemic and central exposure to 5-MeO-DMT (Halberstadt, 2016; Halberstadt et al., 2008, 2012; Jiang et al., 2016b; Shen et al., 2010b). In contrast, chronic treatment with MAO inhibitors suppresses response to 5-MeO-DMT in rodents (Gudelsky et al., 1986b; Lucki and Frazer, 1982).

Potential drug interactions with tetrahydrocannabinol (THC), mitragynine, lithium, haloperidol, benzodiazepines and antidepressants have been investigated in rodents. Small doses of 5-MeO-DMT rescue memory impairments produced by THC (Egashira et al., 2002). Mitragynine suppresses 5-MeO-DMT-induced head-twitch response in mice (Matsumoto et al., 1997). Chronic lithium treatment potentiates the serotonin behavioural syndrome in rats, particularly flat posture and tremor but attenuates head-twitch and ‘wet-dog shake’ response (Goodwin et al., 1986a, 1986b; Harrison-Read, 1979; Kofman and Levin, 1995). Acute benzodiazepine treatment potentiates 5-MeO-DMT-induced head-twitch response (Moser and Redfern, 1988), but attenuates hyponeophagia (Shephard and Broadhurst, 1982). Chronic administration of tricyclic antidepressants consistently attenuates 5-MeO-DMT-induced analgesia (Danysz et al., 1986), head-twitch response (Friedman et al., 1983; Metz and Heal, 1986) and behaviour response (Stolz et al., 1983). However, enhanced responsiveness to 5-MeO-DMT was observed upon 24–48 h withdrawal from the last dose of some tricyclic antidepressants (Friedman et al., 1983; Stolz et al., 1983). Chronic treatment with fluoxetine, a selective serotonin reuptake inhibitor, reduced response to 5-MeO-DMT, which remained attenuated for 3 days following fluoxetine withdrawal (Stolz et al., 1983) and, in a different study, continued to be attenuated until day 9, returning to control levels on day 14 (Rényi et al., 1986b). Citalopram inhibited response to 5-MeO-DMT acutely, but had no effect after 4 h to 7 days (Rényi et al., 1986b). Acute fluoxetine enhanced response to 5-MeO-DMT (Winter, 1999). Chronic haloperidol treatment had no effect on 5-MeO-DMT response (Friedman et al., 1983).

### Toxicology

The LD_50_ in sheep is 1 mg/kg (see Table 2), ranges from 48 to 278 mg/kg in mice (depending on route of administration) (Gillin et al., 1976; Ho et al., 1970) and in cats is 15 mg/kg (Benington et al., 1965).

There have been studies of 5-MeO-DMT toxicity in mice, rats, cats, sheep and monkeys (Benington et al., 1965; Gillin et al., 1976). High doses of 5-MeO-DMT produce ataxia, mydriasis, head nodding, lateral head weaving, tremor, convulsions, shivering, tachycardia and loss of consciousness and in toxic doses respiratory failure (Grahame-Smith, 1971; Lucki et al., 1984).

### Tolerance

Tolerance develops to some (but not all) behavioural and physiological effects of 5-MeO-DMT in rats, cats and monkeys. Studies with once-daily dose regimens reported no tolerance to 5-MeO-DMT-induced changes in neuronal activity in the raphe nucleus (Larson, 1984) or ataxia, decrease in movement and unresponsiveness to loud noise/touch in rhesus monkeys (Gillin et al., 1976). No tolerance was observed in behavioural effects in macaque monkeys administered 0.25 mg/kg IM 5-MeO-DMT every day for 8–12 days. With more frequent drug administration of 0.25 mg/kg IM 5-MeO-DMT administered every 30 min for 9 and 26 h subsequently, tolerance developed to limb jerks, body shakes and checking behaviour and persisted for 26 h (Heinze et al., 1983; Schlemmer and Davis, 1986). Likewise, when 5-MeO-DMT was administered every 30 min for 4 h (at 2 mg/kg IP) to rats, tolerance to the serotonergic behavioural syndrome developed and persisted for 4 h (Trulson and Keltch, 1985). Chronic, frequent administration of 5-MeO-DMT diminishes the responsiveness of 5-HT_1A_ receptor-mediated changes in body temperature and corticosterone secretion without altering the responses mediated by 5-HT_2_ receptors (Nash et al., 1989).

Physical dependence or withdrawal signs have not been reported in any of the repeated dose-administration studies (Gillin et al., 1976; Larson, 1984; Nash et al., 1989; Schlemmer and Davis, 1986; Sills et al., 1985; Trulson and Keltch, 1985).

### Abuse potential and prevalence of use

No studies have investigated whether laboratory animals self-administer 5-MeO-DMT. However, similar studies with other classical psychedelics failed to induce self-administration, or did so only marginally and transiently (Fantegrossi et al., 2004; Yanagita, 1986). There is evidence that 5-HT_2C_ receptor agonists possess anti-addictive properties (Canal and Murnane, 2017).

5-MeO-DMT is not specifically mentioned by the United Nations Office on Drugs and Crime (2020) World Drug Report or the European Drug Report of the European Monitoring Centre for Drugs Drug Addiction (EMCDDA, 2019) or the Global Drug Survey (GDS, 2020; Global Drug Survey, 2021). When it is mentioned, it is often subsumed under the moniker of ‘novel psychoactive substances’, rendering estimation of prevalence of use problematic. A large annual cross-sectional population survey in the United States, National Survey on Drug Use and Health (NSDUH), includes data on 5-MeO-DMT (see Supplementary Information). Over the last 18 years (2002–2019) and 722,653 total respondents aged 12 and older, 33 and 13 respondents (0.0046% and 0.0018% unweighted estimate) reported the lifetime use of 5-MeO-DMT or bufotenine/toad secretions, respectively (Substance Abuse Mental Health Services Administration (SAMHSA), 2021a), and the rates of reporting were steady at 2–3 per year (Palamar and Le, 2019). Because of rarity and possible underreporting, it is difficult to accurately extrapolate prevalence in the general population, but the estimate is around 0.003% for 5-MeO-DMT (Palamar and Le, 2019; SAMHSA, 2021a; Sexton et al., 2020). According to a survey of Australian ecstasy users, only 2% have ever tried 5-MeO-DMT (Bruno et al., 2012).

It is likely appropriate to consider 5-MeO-DMT to have limited abuse liability given anecdotal reports of behaviourally impairing effects (i.e. intoxicating effects that could result in harm) similar to other classic psychedelic compounds (Johnson et al., 2018).

### Epidemiological studies of human recreational/spiritual use

There are no published human clinical trials of 5-MeO-DMT. The published data include a case report of improved outcome measures following sequential administration of ibogaine and 5-MeO-DMT in a veteran with alcohol use disorder (Barsuglia et al., 2018); epidemiological studies and surveys of recreational/spiritual/medicinal use (Barsuglia et al., 2018; Davis et al., 2018, 2019, 2020; Lancelotta and Davis, 2020; Palamar and Acosta, 2020; Uthaug et al., 2019, 2020a, 2020b); and accounts of self-experimentation and recreational/spiritual use (Erowid, 2021; Metzner, 2013; Ott, 2001; Shulgin and Shulgin, 1997).

Reported recreational dose ranges are inhalation: ~6–20 mg; intravenous injection: ~0.7–3.1 mg; sublingual or intranasal routes: ~10 mg; intramuscular: ~5–10 mg; and oral: ~10–30 mg; although Shulgins report it is inactive without a MAO inhibitor (Erowid, 2021; Ott, 2001; Shulgin and Shulgin, 1997). 5-MeO-DMT has a rapid onset when smoked or vapourised: effects peak in 2–5 min, last 15–20 min and return to baseline by 30 min (Davis et al., 2018a). Insufflated, the experience lasts longer, up to 45 min, and the onset is slower (5–7 min) (Metzner, 2013). Users report that smoking/vaporising 5-MeO-DMT elicits more intense effects compared to most other psychedelics (Barsuglia et al., 2018; Davis et al., 2018). Although no qualitative studies so far directly compared phenomenology of 5-MeO-DMT-elicited experience with other short-lasting psychedelics frequently referred to as intense, such as DMT or *Salvia divinorum*, anecdotal reports describe that 5-MeO-DMT feels very distinct. The subjective experience is generally described as transcendent, often involving ego-dissolution, non-dual awareness and an increased range and intensity of emotions, spanning the feeling of love, unity and awe to panic and terror. Notable is the frequent absence of visual effects (Erowid, 2021). It is possible that the absence of visual effects is due to 5-HT_1A_ receptor action, as it was demonstrated that 1A receptor agonists reduce visual imagery induced by psilocybin (Pokorny et al., 2016). In contrast to highly detailed DMT or salvia trips, users of 5-MeO-DMT often describe content-free experiences, associate with loss of sense of self and bodily awareness, and sensory deprivation (described as all-white light, or all-black), with common descriptors such as: ‘emptiness’, ‘nothingness’ or ‘void’ (Millière et al., 2018). Dose, set and setting have considerable impact on the perceptual and emotional experience and, in common with all psychedelics, adequate preparation has been reported to be important (Lancelotta and Davis, 2020; Metzner, 2013). Anecdotal reports and surveys indicate that repeated dosing with 5-MeO-DMT is possible, with almost no desensitisation or tolerance to psychedelic effects reported (Davis et al., 2018; Trout, 2007; Uthaug, 2020a, 2020b).

Retrospective surveys examined 5-MeO-DMT patterns of use, motivations for consumption, subjective effects and potential benefits and consequences associated with use. It is worth noting that survey data is likely biased towards positive outcomes due to selection bias.

The main reasons for trying 5-MeO-DMT were spiritual exploration (68%), recreation (18%) or healing (14%); most people used it less than 4 times in their life (59%). 90% reported positive and/or transcendent experiences, 57% fit the criteria for complete mystical experience (scored as reaching ⩾60% on each of the subscales of the Mystical Experience Questionnaire, MEQ-30; Barrett et al., 2015) with around 37% having challenging experiences (measured by the Challenging Experience Questionnaire; Barrett et al., 2016; Davis et al., 2018a).

In a subsequent survey, Davis et al. (2019) collected self-report measures of depression and anxiety in 362 people who took 5-MeO-DMT in a group setting. Of those diagnosed with depression (41%) or anxiety (48%), most reported these conditions were improved (depression = 80%; anxiety = 79%) following 5-MeO-DMT use, and fewer reported they were unchanged (depression = 17%; anxiety = 19%) or worsened (depression = 3%; anxiety = 2%). Associations were reported between improvement in depression/anxiety, and greater intensity of mystical experiences (as measured by MEQ-30) and higher ratings of the spiritual significance/personal meaning of the 5-MeO-DMT experience (Davis et al., 2019). Moreover, supportive setting in a group was associated with much higher ratings of complete mystical experience – 83%, compared to 54% of respondents who had 5-MeO-DMT experience in the recreational setting, and inverse relationship was noted for challenging experiences (Sepeda et al., 2019).

Several countries where 5-MeO-DMT is unregulated offer retreats and treatment programmes. A survey of 51 US Special Operations Forces Veterans from one such retreat, with combined 5-MeO-DMT and ibogaine treatments, indicated the experience was therapeutic for their traumatic experiences, suicidal ideation, depression and anxiety (Davis et al., 2020). Another case study presents brain imaging data from one participant (31-year-old military veteran with alcohol use disorder) of a similar treatment centre in Mexico (Barsuglia et al., 2018). Single-photon emission computed tomography (SPECT) neuroimaging after treatment with ibogaine and 5-MeO-DMT showed increases in brain perfusion in bilateral caudate nuclei, left putamen, right insula, as well as temporal, occipital and cerebellar regions compared to baseline. The patient reported improvement in mood, cessation of alcohol use and reduced cravings at 5 days post-treatment, effects which were sustained at 1 month, with a partial return to mild alcohol use at 2 months (Barsuglia et al., 2018). In a survey of 20 individuals from the same retreat centre, 75% reported a ‘complete mystical experience’, as measured by MEQ-30 (Barsuglia et al., 2018).

Two prospective studies examined the effects of vapourised 5-MeO-DMT inhalation (11 participants) (Uthaug et al., 2020b) and the effects of toad secretions (42 participants) (Uthaug et al., 2019). In both studies, compared to baseline, the ratings of mindfulness facets increased (measured with Five Facets Mindfulness questionnaire, FFMQ-15; Gu et al., 2016), while ratings of depression and anxiety decreased (measured with Depression, Anxiety and Stress scale, DASS-21 (Henry and Crawford, 2005) or with Brief Symptom Inventory, BSI-18 (Derogatis, 2001)) immediately after the session and remained so at follow up. Whether there are any potential clinical implications of this is unclear.

Acute adverse effects of 5-MeO-DMT reported in some of the above studies (Barsuglia et al., 2018; Davis et al., 2018, 2019; Uthaug et al., 2020b) include fear, sadness, anxiety, confusion, profound experience of one’s own death, crying, paranoia, shaking/trembling, vomiting, nausea, transient headache, pressure or weight in the chest or abdomen and loss of body perception (Table 4). Dissociative experiences with memory loss (blackout) have been reported (Metzner, 2013). Delayed adverse effects (up to 1 week) included somatic tension in muscles, difficulties sleeping, ‘flashbacks’ or ‘reactivations’ – re-experiencing some of the effects felt during the drug session (Uthaug et al., 2020a, 2020b), and in rare cases – psychosis (Metzner, 2013; Sauras Quetcuti et al., 2019; Shulgin and Shulgin, 1997). In an online retrospective survey, flashbacks were reported as more common with higher doses and with vaporised rather than intramuscular administration (Uthaug et al., 2020a).

**Table 4.** Adverse effects of 5-MeO-DMT from human epidemiological studies and published ‘underground’ reports.

Study	Acute adverse effects	Delayed adverse effects
Davis 2020 (retrospective survey)	Participants reported that psychedelic treatment they received (ibogaine plus 5-MeO-DMT) was one of the most psychologically challenging (69%) experiences of their entire lives Adverse events not assessed	Not assessed
Palamar and Acosta (2020; retrospective survey)	Not assessed	Not assessed
Uthaug (2020b) (prospective study of synthetic 5-MeO-DMT in naturalistic settings)	45.5% (*N* = 5) of the sample reported adverse effects post-session. One participant reported feeling ‘scared and confused’, one participant reported ‘feeling anger, joy love and fear’, one participant vomited shortly after intake, one participant expressed ‘feeling a little shock on the first try, but nothing bad’ and finally one participant reported feeling that their throat was scratching from smoking	On the 7-day follow-up, 27.3% (*N* = 3) of the sample reported adverse effects in the days following the session. One participant reported some affective symptoms and somatic tension in muscles, one participant reported difficulties sleeping (insomnia), and one participant reported experiencing somatic tension in muscles
Uthaug (2020a) (retrospective survey)	Not assessed	‘Reactivation’ or flashback experiences reported more common with vaporised route of administration compared to the intramuscular (3/14 vs 9/13 participants, respectively)
Uthaug (2019) (prospective study of toad secretions in naturalistic setting)	Not assessed	Not assessed
Davis 2019 (retrospective survey)	Assessed using CEQ: *M*_intensity_ = 0.8 (SD = 0.8), range 0–5. Subscales: Isolation = 0.4(0.9) Fear = 1.0(1.3) Grief = 0.9(1.1) Physical Distress = 0.9(0.9) Insanity = 0.5(1.0) Death/Dying = 1.5(1.7) Paranoia = 0.0(0.3) There were no differences in the intensity of acute challenging experiences between those who did or did not report an improvement in depression or anxiety, which could be because respondents reported only a ‘slight’ intensity of challenging experiences	Not assessed
Barsuglia et al. (2018) (retrospective survey)	Not assessed	Not assessed
Barsuglia et al. (2018) (case study)	Physical purging through dry heaving that lasted for several minutes	Not assessed
Davis et al. (2018) (retrospective survey) Lancelotta and Davis (2020) (same survey)	Assessed using CEQ: *M*_intensity_ = 0.95, SD = 0.91; range 0–5. Subscales: Isolation = 0.76(1.23) Fear = 1.22(1.38) Grief = 0.69(1.00) Physical Distress = 1.15(1.09) Insanity = 0.85(1.21) Death/Dying = 1.75(1.90) Paranoia = 0.18(0.60) On average 37% of respondents reported experiencing challenging psychological and somatic experiences. Between 40% and 66% reported experiences of feeling their heart beat, fear, frightened, their body shake/tremble, anxious, as if they were dead or dying, shaky inside, that something horrible would happen, like crying, pressure or weight in their chest or abdomen, and panic, and having the profound experience of their own death	Not assessed
Metzner (2013) (qualitative field report of ‘underground’ use)	‘Dissociative experiences’, involving losing consciousness and memory of the drug session, psychotic or fear-panic reactions occurred in about 10% of cases. Most dissipate as the drug wears off	Flashback/reactivation experience (of the dissociative, fear or psychotic reactions)
Ott (2001) (self-experimentation)	Tinnitus	Not reported
Shulgin and Shulgin (1997) (qualitative field reports of ‘underground’ use and self-experimentation)	Nausea, tinnitus, fear, feeling like dying, blackout, purple face and no breathing (in one case with unknown but very large smoked dose)	Psychosis, terror, lack of sleep (one report)

SD: standard deviation.

In the published mortality and morbidity reports mentioning 5-MeO-DMT, it had been taken as toad secretions, concurrently with other drugs of abuse or together with monoamine oxidase inhibitors (MAOIs). One of the earliest toxicity reports is of a 5-year-old child hospitalised with profuse salivation and continuous seizures after licking *Incilius alvarius* toad (Hitt and Ettinger, 1986). A 17-year old was hospitalised with extreme agitation, hyperthermia, tachycardia and rhabdomyolysis after consuming 5-MeO-DMT and the MAOI harmaline (Brush et al., 2004). There are reported fatalities, including a 25-year old who ingested ayahuasca with 5-MeO-DMT (Sklerov et al., 2005).

There are no reports of deaths related to 5-MeO-DMT from the Office for National Statistics (2020) (England and Wales), DAWN 2011 report/2020 preliminary data (Drug Abuse Warning Network, SAMHSA, 2021b), the Report of the American Association of Poison Control Centers’ National Poison Data (Gummin et al., 2020) or the National Programme on Substance Abuse Deaths (NPSAD, 2021). This could be because use is still relatively limited, the toxicity is very low, this substance is not routinely tested for and/or because 5-MeO-DMT is not included in most national databases or epidemiological surveys (Palamar and Le, 2019).

## Discussion

In this review we have summarised and synthesised the data on 5-MeO-DMT thus far to inform controlled clinical trials of its basic safety, pharmacokinetic and pharmacodynamic profiles in humans.

5-MeO-DMT is a naturally occurring tryptamine derivative found in gland secretions of the Sonoran Desert toad, in a variety of plants, and endogenously in mammals. It is also available as a pure compound. There have been no published laboratory studies in humans on the effects of 5-MeO-DMT, except for a case study with a single individual (Barsuglia et al., 2018).

Animal studies have demonstrated paradoxical (non-linear or biphasic) effects of 5-MeO-DMT on pharmacokinetics, thermoregulation, nociception, heart rate and blood pressure. Dose finding studies with different routes of administration of 5-MeO-DMT in humans are required to establish the therapeutic dose range and safety profile. The pharmacokinetic profile of the therapeutic dose range in humans needs to be determined, as studies with rodents indicate that higher doses result in non-linear PK profile, and subjective effects at higher doses might depend on the CYP2D6 genotype.

The available data indicate that established safety measures for psychedelic research should be implemented for 5-MeO-DMT human clinical trials. Concomitant use of MAOIs and lithium should be avoided. Flashbacks or ‘reactivations’ have been reported in surveys of recreational use of psychedelics including 5-MeO-DMT. Such effects have not been observed in clinical studies of psychedelics to date indicating the importance of screening, monitoring and other safety measures. Hallucinogen-persisting perception disorder (HPPD) describes a nebulous set of symptoms persisting weeks, months or years after psychedelic use that are associated with anxiety or distress. The prevalence of HPPD is estimated to be very rare among classical psychedelic users (Halpern et al., 2018), with one estimate being that it is present in 1 in 50,000 psychedelic users (Grinspoon and Bakalar, 1998). The clinical concept is sufficiently vague to make true estimate of prevalence very difficult. This is further complicated by recreational users taking psychedelics with other drugs including alcohol. An analysis of people reporting symptoms of HPPD found that symptoms were more frequently preceded by use of non-psychedelic substances such as alcohol, tobacco and cannabis than by the use of psychedelics and that some individuals with these symptoms had never taken a psychedelic (Halpern et al., 2018). These data call into question whether HPPD is peculiar to psychedelic use and suggest that it may instead constitute a syndrome aetiologically related to many different psychoactive substances, occurring in those with a pre-existing vulnerability. A direct neurotoxic effect appears unlikely.

The therapeutic potential of 5-MeO-DMT is hypothetical, but intriguing. Surveys of recreational users suggest rapid anxiolytic and antidepressant properties not dissimilar to those being probed in early-phase studies of psilocybin and LSD, as well as later phase studies of ketamine and its analogues. 5-MeO-DMT shares similar pharmacology to other classical psychedelics; however, the specific pharmacokinetic and pharmacodynamic properties of the drug may confer clinical advantages. One of these is a short duration of action, which may require less healthcare resource utilisation and thus increasing access to treatment. Another is the absence of visual effects, which could be distracting. Their absence might lead to higher rates of mystical experiences. As such, it deserves further investigation as a putative rapid-acting antidepressant. A key step will be establishing a pharmacokinetic profile and safety profile of 5-MeO-DMT in healthy volunteers in a controlled trial design.

## Conclusion

5-MeO-DMT is a short-lasting psychedelic substance with a unique subjective effect profile making it an intriguing compound to research. The available data indicate the risk profile of 5-MeO-DMT is similar to other classic psychedelics, such as psilocybin and that established safety precautions for psychedelic research be followed. A notable feature of 5-MeO-DMT is the reportedly high rates of the ego-dissolution and mystical experiences, which in studies with other psychedelics are related to long-term positive therapeutic outcomes, calling for clinical exploration.

## Supplemental Material

sj-docx-1-jop-10.1177_02698811211050543 – Supplemental material for A narrative synthesis of research with 5-MeO-DMT Click here for additional data file. Supplemental material, sj-docx-1-jop-10.1177_02698811211050543 for A narrative synthesis of research with 5-MeO-DMT by Anna O Ermakova, Fiona Dunbar, James Rucker and Matthew W Johnson in Journal of Psychopharmacology
