# Supplementary material for: A narrative synthesis of research with 5-MeO-DMT
Source: J Psychopharmacol. 2021 Oct 19;36(3):273–94. doi: 10.1177/02698811211050543 (PMC8902691; doi:10.1177/02698811211050543)
Supplement: sj-docx-1-jop-10.1177_02698811211050543 – Supplemental material for A narrative synthesis of research with 5-MeO-DMT [file sj-docx-1-jop-10.1177_02698811211050543.docx]

**Supplementary material**

**S1: PubMed search terms**

methoxydimethyltryptamines[MeSH Terms] OR "methoxydimethyltryptamines"[All Fields] OR "5 meo dmt"[All Fields] OR ("methoxydimethyltryptamines"[MeSH Terms] OR "methoxydimethyltryptamines"[All Fields] OR "5 methoxy n n dimethyltryptamine"[All Fields]) OR "5-methoxy-dimethyltryptamine"[All Fields] OR ("Bufo"[All Fields] AND "alvarius"[All Fields]) OR ("Incilius"[All Fields] AND "alvarius"[All Fields])

**S2: National Survey on Drug Use and Health: Prevalence of lifetime self-reported use of NPS from concatenated data 2002-2019**

**About the survey**

Data came from 18 most recent cohorts of National Survey on Drug Use and Health (NSDUH 2002-2019). NSDUH is an annual cross-sectional population survey in the 50 states and District of Colombia (Substance Abuse and Mental Health Services Administration [SAMHSA]). NSDUH is a nationally representative probability sample of populations aged older than 12, living in households (excluding institutionalised individuals).

NSDUH provides sampling weights to address unit- and individual-level non-response. Weights are adjusted to ensure estimates are consistent with estimates provided by the US Census Bureau. Further information on sampling and survey techniques can be found elsewhere ([SAMHSA, 2021](https://www.datafiles.samhsa.gov/sites/default/files/field-uploads-protected/studies/NSDUH-2002-2019/NSDUH-2002-2019-datasets/NSDUH-2002-2019-DS0001/NSDUH-2002-2019-DS0001-info/NSDUH-2002-2019-DS0001-info-codebook.pdf)). The weights measure the number of people in the population that are represented by each member of the sample.

We have only looked at the questions related to hallucinogen use explained in the NSDUH as “questions are about substances called hallucinogens. These drugs often cause people to see or experience things that are not real.” NSDUH then provided a list of “common hallucinogens” and note that “these and many other substances that people use as hallucinogens are often known by street names, and we can't list them all.” They then instruct the respondent to look at the listed drugs (LSD, mescaline, peyote, psilocybin, PCP, ecstasy). Respondents were then asked, “Have you ever, even once used any other hallucinogens besides the ones that have been listed?” If the respondent checked of “yes” they were then given the opportunity to type in names of up to five drugs (“Please type in the name of the other hallucinogens you have used”). If respondents were not sure how to spell the drug’s name, they were instructed to make their “best guess”. NSDUH provided five variables in its public dataset, each containing coded drug name response categories (containing the number of subjects who typed that drug name in); e.g., category response #6061 was labelled “5-MeO-DMT” and #606 “Bufotenin, toad, toad licking” to indicate that the subject typed in the name of this particular drug.

**Results**

A total of 14.57% of the total sample reported ever using hallucinogens. Of the total sample only 1.47% reported using ‘unusual’ hallucinogens (i.e. psychedelics other than LSD, mescaline, peyote or psilocybin), corresponding to unweighted sample of 13,977 people (and 18-year weighted n=2,651,864). Of those respondents indicating they’ve used other psychedelics, 33 and 13 indicated they’ve tried 5-MeO-DMT and toad/bufotenine respectively, which is less than 0.003% of the total population.

Ever used any hallucinogens

|  | **Unweighted** | **18-y weighted** | **% Total (weighted)** |
| --- | --- | --- | --- |
| Total | 722,653 | 180,602,641 | 100.00% |
| Never used | 621,593 | 154,286,920 | 85.43% |
| Ever used | 101,060 | 26,315,721 | 14.57% |

Ever used other hallucinogens (NOT LSD, mescaline, peyote, psilocybin, PCP)

|  | **Unweighted** | **18-y weighted** | **% Total, weighted** |
| --- | --- | --- | --- |
| Total | 722,653 | 180,602,641 | 100.00% |
| Yes | 13,977 | 2,651,864 | 1.47% |
| No | 88,368 | 23,835,514 | 13.20% |
| Never used hallucinogens | 619,338 | 153,941,997 | 85.24% |
| Don’t know | 848 | 144,463 | 0.08% |
| Missing | 113 | 26,632 | 0.01% |

**Summary table**

|  | **Unweighted** | | | **18-year weighted** | | |
| --- | --- | --- | --- | --- | --- | --- |
|  | **Users (N)** | **% in subsample “other hallucinogens”**  **(N = 13,977)** | **% of full sample (N = 722,653)** | **Users (N)** | **% in subsample “other hallucinogens”**  **(N = 2,651,864)** | **% of full sample (N = 180,602,641)** |
| **5-MeO-DMT** | 33 | 0.2361% | 0.0046% | 5,115 | 0.1929% | 0.0028% |
| **Bufotenine/ Toad** | 13 | 0.0930% | 0.0018% | 2,521 | 0.0951% | 0.0014% |

The values presented were derived from 18 years of data for all subjects’ lifetime use of other hallucinogens. Respondents were given the opportunity to type in names of other hallucinogens they used (in addition to LSD, peyote, psilocybin, mescaline, PCP and ecstasy that were asked as separate questions). Since subjects could have reported up to 5 different ‘other hallucinogens’, we added the numbers for five of those for 5-MeO-DMT and toad/bufotenine options.
